# Florivory and Pollination Intersection: Changes in Floral Trait Expression Do Not Discourage Hummingbird Pollination

**DOI:** 10.3389/fpls.2022.813418

**Published:** 2022-03-30

**Authors:** Priscila Tunes, Stefan Dötterl, Elza Guimarães

**Affiliations:** ^1^Postgraduate Program in Biological Sciences (Botany), Institute of Biosciences, São Paulo State University, Botucatu, Brazil; ^2^Laboratory of Ecology and Evolution of Plant-Animal Interactions, Institute of Biosciences, São Paulo State University, Botucatu, Brazil; ^3^Department of Environment and Biodiversity, Paris Lodron University of Salzburg, Salzburg, Austria

**Keywords:** floral colour, floral damage, floral scent, hummingbird pollination, *Pyrostegia venusta*, floral cues, plant-pollinator communication

## Abstract

Many flowers are fed on by florivores, but we know little about if and how feeding on flowers affects their visual and chemical advertisement and nectar resource, which could disrupt pollination. Here, we investigated if damages caused by florivores compromise a Neotropical hummingbird pollination system, by modifying the floral advertisements and the nectar resource. We surveyed natural florivory levels and patterns, examined short-term local effects of floral damages caused by the most common florivore, a caterpillar, on floral outline, intra-floral colour pattern and floral scent, as well as on the amount of nectar. Following, we experimentally tested if the most severe florivory pattern affected hummingbird pollination. The feeding activity of the most common florivore did not alter the intra-floral colour pattern, floral scent, and nectar volume, but changed the corolla outline. However, this change did not affect hummingbird pollination. Despite visual floral cues being important for foraging in hummingbirds, our results emphasise that changes in the corolla outline had a neutral effect on pollination, allowing the maintenance of florivore–plant–pollinator systems without detriment to any partner.

## Introduction

Our knowledge is growing fast concerning the visual and chemical signal diversity involved in plant–pollinator communication (Leonard and Masek, 2014; Schaefer and Ruxton, 2015; Kantsa et al., 2017; Leonard and Francis, 2017). However, we know little about if and how florivores, by feeding on flowers, affect these signals and consequently the visual (Johnson et al., 1995; Krupnick et al., 1999) and chemical advertisements (McCall and Irwin, 2006; Lucas-Barbosa et al., 2011; Späthe, 2013; Vega-Polanco et al., 2020), which could disrupt the communication between flowers and pollinators. Indeed, there is accumulated evidence that pollinators do respond to damages caused by florivores, for example, performing less visits (Krupnick et al., 1999; Mothershead and Marquis, 2000; Pohl et al., 2006; McCall, 2008; Tsuji et al., 2016; Muola et al., 2017), which has negative effects on pollination success (Kessler and Halitschke, 2009; Haas and Lortie, 2020).

The effects of florivore feeding can be local, restricted to a single flower, and/or systemic, involving the activation of phytohormonal signaling pathways (Chrétien et al., 2018; Rusman et al., 2019a). So far, most studies have focused on the outcome of florivory on plant fitness (Haas and Lortie, 2020; Boaventura et al., 2022). There is, however, limited knowledge regarding which specific changes in floral advertisement, being them locally or systemically elicited, act as triggers for behavioral responses displayed by pollinators. Indeed, we do not know if florivore-induced changes in a single floral cue are enough to affect pollinator behavior in natural systems or if changes in multiple floral cues are required, as pollinators usually integrate between visual and olfactory cues (Leonard and Masek, 2014; Junker and Parachnowitsch, 2015).

Among such pollinators that use both visual and olfactory cues when looking for flowers are hummingbirds (Altshuler and Nunn, 2001; Hurly and Healy, 2002; Kessler et al., 2008). They are highly specialised pollinators in the Neotropics (Stiles, 1981; Zanata et al., 2017) with a well-developed visual system (Hickman and Robert, 1993; Pritchard et al., 2017; Tyrrell et al., 2018 and references therein). Indeed, there is experimental evidence that hummingbirds recognise specific floral shapes and prefer visiting those associated to their bill shape and size (Maglianesi et al., 2015; Rico-Guevara et al., 2021). As florivory implies damage to the flowers, which affects flower integrity and shape, we expect that these changes *per se* could be enough to interfere in the visual communication between flowers and hummingbirds, causing hummingbirds to neglect damaged flowers. Even though there was a long-held belief that olfaction is not involved in the location and selection of flowers by hummingbirds, it is meanwhile known that they can perceive (Steiger et al., 2008; Wester and Lunau, 2017) and respond (Kessler et al., 2008) to floral volatile compounds. By feeding on floral tissue that is potentially involved in the biosynthesis and emission of floral scent, florivores might affect the total amount of scent emitted by flowers (Zangerl and Berenbaum, 2009; Tunes and Guimarães, 2020) and the chemical composition of floral scent (Lucas-Barbosa et al., 2013; Farré-Armengol et al., 2015). Moreover, these florivory-induced changes in floral scent can be expressed exclusively locally, on the damaged flower itself, or systemically, leading to changes in the damaged flower as well as in undamaged flowers of the same plant (Dicke et al., 1990; Turlings and Tumlinson, 1992; Potting et al., 1995; Röse et al., 1996; Lucas-Barbosa et al., 2013). In addition to the advertisement, florivory might also have effects on floral resources. Although it is not expected that florivores deplete the nectar resource used by hummingbirds, since they often feed on floral tissues other than nectaries and not on nectar itself (Xiao et al., 2021), the injuries caused by florivores could interfere with nectar secretion by altering plant physiological pathways. In fact, by feeding on plant tissues, herbivores can alter jasmonate pathways, which are involved in both plant defense (Xie et al., 1998; Chrétien et al., 2018) and nectar secretion (Radhika et al., 2010). Therefore, we considered that florivores could act indirectly on floral nectar resources, beyond merely acting on floral advertisements. Thus, as flowers are the pollination units that are recognised and pursued by pollinators, direct and indirect changes that happen during a flower lifetime might have significant consequences for the pollination process. In this study, we focused on short-term local effects of florivory on floral advertisement, floral resource, and pollination. Specifically, we aimed to elucidate if florivory affects the visual and olfactory advertisement, the nectar resource, and finally the pollination success in a hummingbird-pollinated plant. Therefore, we investigated (i) natural florivory levels and florivores (ii) if and which florivory-induced local changes occur in floral visual and olfactory advertisements, and (iii) if florivory affects nectar volume. Finally, (iv) we simulated florivory-induced floral changes and experimentally tested if they discourage hummingbird pollination.

## Materials and Methods

### Focal Plant Species, Its Pollinators, and Study Site

**Figure 1:**
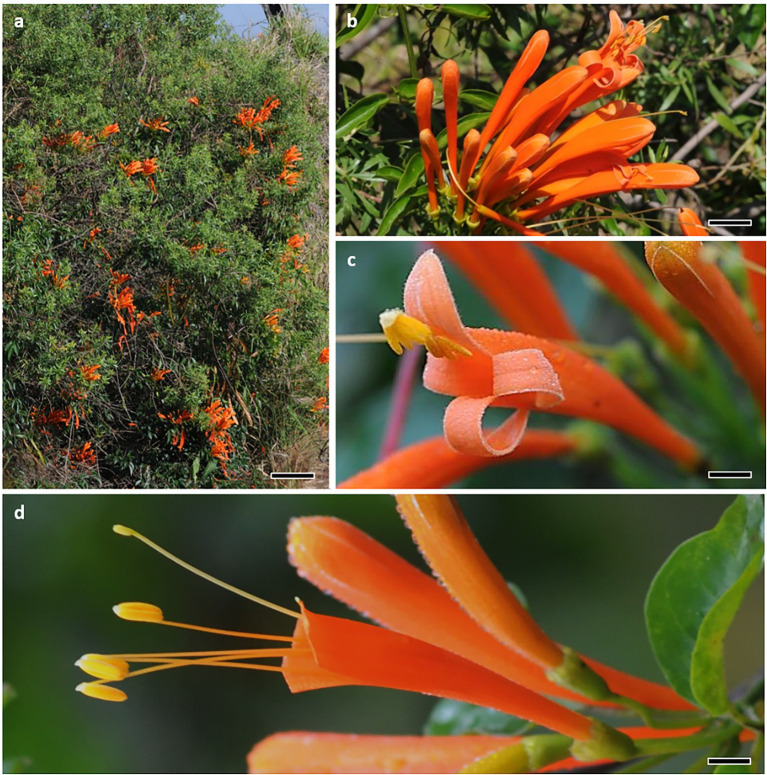
*Pyrostegia venusta*. **(A)** Flowering plant. Scale bar: 20 cm. **(B)** Inflorescence with undamaged open flowers and flower buds. Scale bar: 2.5 cm. **(C)** Recently opened flower. Scale bar: 0.8 cm. **(D)** Natural floral damage corresponding to 51–60% of the corolla. Scale bar: 0.5 cm.

*Pyrostegia venusta* (Ker-Gawl.) Miers (Bignoniaceae) is a neotropical vine (Figure 1A) that occurs from the northeast coast of Brazil to the northeast of Argentina (approximately 35°–58° W and 5°–30° S; Pool, 2008). The flowering period of this plant species occurs from April to October, with a peak in July/August (our study period), when there was the highest number of flowers opening per day (Rossatto and Kolb, 2011). This species shows terminal or axillary panicles (Figure 1B); zygomorphic flowers with a long tubular orange corolla presenting curved-back lobes; four exerted didynamous stamens; a syncarpous gynoecium with bilocular ovary, long style, and bilabiate stigma (Figure 1C) with lobes that close following mechanical stimulation (Pool, 2008; Singh et al., 2009). Flower anthesis starts at approximately 06:30 h and lasts for approximately 48 h (Galetto et al., 1994). *Pyrostegia venusta* is self-compatible (Gobatto-Rodrigues and Stort, 1992), with hermaphroditic and protandrous flowers (Pool, 2008) that show diverse degrees of approach herkogamy. Most flowers present the gynoecium longer than the androecium, whereas others do not (Pool, 2008; Tunes and Guimarães, 2020). We deposited a voucher specimen in the Herbarium BOTU ‘Irina Delanova de Gemtchujnicov’ (voucher number 30788). The authorisation for collection of biological samples is registered on Sisgen under #A90A83C.

Floral nectar is sucrose-rich, with a concentration of 28% of sugars (Galetto et al., 1994). It is consumed by hummingbird pollinators, such as *Eupetomena macroura*, *Phaethornis squalidus*, *P*. *pretrei* (Leone, 2011), *Chlorostilbon lucidus* (previously *C*. *aureoventris* (Grantsau, 1988)), and *Sappho sparganura* (Galetto et al., 1994). Even though this plant species is self-compatible, and some flowers are capable of autonomous selfing and of being self-pollinated by small bees, hummingbird pollination is crucial for plant reproduction, especially when considering that most of the flowers have approach herkogamy and only hummingbirds are capable of transferring pollen among those flowers (Tunes and Guimarães, 2020).

This study was performed in a natural population growing at the edge of a seasonal tropical forest fragment (at 22°53’ S and 48°29’ W), in Botucatu municipality, São Paulo state, Brazil. To evaluate the effects of florivory on floral traits and to assess the effect of experimentally simulated floral damages on hummingbird pollination, we randomly assigned different individuals to the investigation of each trait (floral colour pattern, floral scent, floral nectar) and for the field experiment, totaling 62 different plants.

### Natural Incidence of Florivory and Florivores Associated to It

We randomly designated *P*. *venusta* plants to assess the percentage of flowers with natural damages to the corolla. We sampled all the first- and second-day flowers from 80% of the plants in the population (*n* = 935 flowers, from 58 plants). Then, we visually estimated the percentage of corolla removal in each damaged flower and classified the flowers into one of the following categories (adapted from Dirzo and Domingues, 1995): 1–3%, 4–5%, 6–10%, 11–20%, 21–30%, 31–40%, 41–50%, and 51–60% of removed tissue. Additionally, we performed 40 h of flower observations in ten different days, during the morning (15 h), afternoon (15 h), and night (10 h), to identify the animals responsible for each type of damage registered on the flowers.

### Evaluating the Effects of Natural Florivory on Floral Traits

We evaluated the effect of the most severe damage (category of 51 to 60% of corolla removal) caused by a caterpillar (the most common florivore) on floral outline, intra-floral colour pattern and floral scent, as well as on the amount of nectar.

#### Floral Outline

Floral outline (*sensu* Herrera, 1993) may act as a signal from the perspective of a hummingbird when hovering in front of a flower. Florivores, by feeding on flowers, have the potential to change the floral frontal outline. Here, we investigate if the most common florivore, by feeding on flowers, changes the frontal floral outline, which could lead hummingbirds to neglect damaged flowers.

#### Intra-floral Colour Pattern

When hummingbirds are hovering in front of *P*. *venusta* flowers, they see the yellowish anthers and stigma against the inner surface of the orange upper petal lobes of the corolla, which act as background (Figure 1C). However, when the upper portion of the corolla is removed by florivores, the hummingbirds will see the anthers and stigma against the inner portion of the remaining lower half of the corolla tube (Figure 1D). Thus, in damaged flowers, a different flower portion acts as the background for the reproductive structures. Thus, to accurately determine if florivory changes intra-floral colour pattern, we associated the human-visible colour patterns with UV photography from five intact and five damaged flowers (*n* = 5 plants, each containing both types of flowers), which allowed us to see if there was any UV pattern that could be modified by florivory. After stablishing the floral portions/structures responsible for creating the intra-floral colour patterns, we measured the spectral reflectance of the anthers, the stigma, the upper petal lobes, and the inner portion of the lower half of the corolla tube (Supplementary Figure 1) of 20 flowers (*n* = 8 plants). Firstly, we measured the reflectance of the stigma, anthers, and superior corolla lobes of undamaged flowers. Then, we manually caused the damage in the same flowers (the same severe damage caused by the most common florivore and used for the pollination experiments, Figure 1D) and measured the reflectance of the inner portion of the lower half of the corolla tube. This is the portion of the corolla that will contrast against the stigma and anthers in the damaged flowers. For UV photography, we used a camera with a modified sensor and lens (Canon EOS Rebel T3i with a 50 mm lens) that only captures UV light from 340 to 400 nm. We illuminated the flowers with a hand-held UV light source, which emits light from 315 to 405 nm, which corresponds to the spectral sensitivity of birds’ UV photoreceptors (Herrera et al., 2008; Ödeen and Håstad, 2010). For spectral reflectance, we used a spectrophotometer (Ocean Optics Jaz-EL200 UV–VIS) and collected reflectance data from 300–700 nm. A deuterium–halogen lamp, that emitted light in the range of 215 to 1700 nm, was used as the light source. We used the reflectance of a ‘diffuse Spectralon reflectance standard’ as white standard and the reflectance obtained from inside a black chamber as black standard, according to Lunau et al. (2011). We took all measurements at 45° in relation to the flower floral surface.

To provide a graphical representation of floral colour as perceived by hummingbirds, we calculated the colour loci of each floral part in the tetrahedron colour space model (Vorobyev et al., 1998). We used the D65 standard daylight illumination (Wyszecki and Stiles, 1982) and a standard function of green leaves (AV 400) as the background. To display the contrast as seen by hummingbirds in intact flowers, we calculated the chromatic and achromatic contrasts of the anthers and of the stigma against the upper petal lobes. To display the contrast in damaged flowers, we calculated the chromatic and achromatic contrasts of the anthers and of the stigma against the upper internal portion of the corolla tube. To evaluate if hummingbirds could perceive the contrast between these floral parts, we considered 1.00 just noticeable differences (JNDs) as a minimum threshold for colour discrimination by hummingbirds (Vorobyev et al., 1998). Thus, any contrast higher than 1.00 JND was considered as perceivable by hummingbirds. We used R v. 4.0.2 (R Core Team, 2020) with the additional packages pavo (Maia et al., 2019) and rgl (Adler, 2020) to create and plot the tetrahedron colour space model and to calculate the chromatic and achromatic contrasts of the stigma and of the anthers against the corolla, as perceived by hummingbirds.

#### Floral Scent

**Figure 2:**
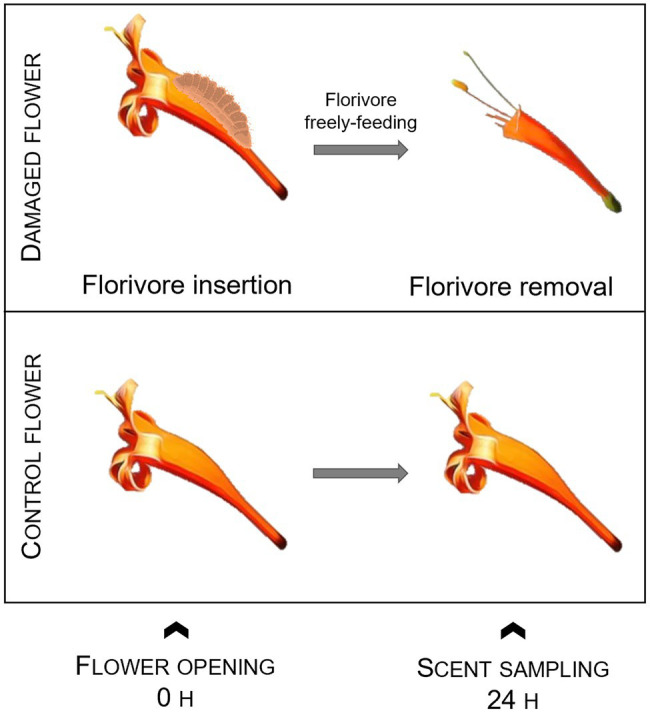
Experimental design used to investigate short-term local changes in floral scent due to florivory by a lycaenid caterpillar. Caterpillar image from Biorender.com.

We verified if florivory by the most common florivores in nature affects local scent emission in terms of the total amount of floral scent, the relative scent composition, and qualitative scent properties during the flower lifespan. Therefore, we bagged intact pre-anthesis buds to ensure that pollinators or other florivores could not visit them. When the flowers opened, we inserted in one flower per plant a florivore into the bag. We kept the florivores inside the bags, freely feeding on the flowers overnight. In the morning of the next day, we removed them and sampled the VOCs (volatile organic compounds) emitted by the flowers (*n*_damaged_ = 5 flowers from 5 different plants). The other flowers (*n*_control_ = 5 flowers from 3 different plants), which remained bagged throughout the experiment, served as positive control, and the emitted scents were also sampled in the morning of the second day (Figure 2). The florivores used in this experiment were Lycaenidae caterpillars, which were the most common florivores found in this system. We collected caterpillars at third and fourth instars from other individuals of the same population and stored them for a few hours in voile bags before transferring them to the flowers to start the experiment.

We collected floral and vegetative (used as negative control) VOC samples following the protocol by Dötterl et al. (2005). We enclosed the flowers or leaves in 12 × 8 cm polyethylene oven bags 10 min before sampling. The VOCs that accumulated inside a bag were collected for 1 h in an adsorbent trap connected to a membrane pump that generated an airflow of 200 ml/min. The adsorbent traps were made from quartz microtubes with approximately 15 mm of length by 2 mm of internal tube diameter. These traps were filled with a mixture of 1.5 mg Tenax-TA (60–80 mesh) and 1.5 mg of Carbotrap B (20–40 mesh, both Supelco). We stored the samples at −20°C until analysis. We analysed the VOC samples on a TD-20 automated thermo desorption system (Shimadzu) coupled to a QP2010 Ultra EI GC/MS (gas chromatograph coupled to a mass spectrometer, Shimadzu) equipped with a ZB-5 fused silica column (60 m long, 0.25 mm of inner diameter, 0.25 μm of film thickness) and maintained a constant 1.5 ml/min flow of helium as the carrier gas. The injector temperature was 200°C and the samples were injected in split mode 1:3. The oven temperature started at 40°C, then increased by 6°C/min to 250°C, at which it kept constant for 1 min. The MS interface was set at 250°C. Mass spectra were taken at 70 eV (in EI mode), with a scanning range of 30–350 m/z. The data were analysed using the GCMSolution package, Version 4.41 (Shimadzu). We performed the tentative identification of the volatile compounds present in each sample by comparison of Kovats retention indices (KRI, based on a series of n-alkanes) and mass spectra to data available in the databases ADAMS (Adams, 2007), ESSENTIALOILS-23P, FFNSC 2, Wiley 9 and Nist11. If possible, compound identities were confirmed by authentic reference standards available at the Plant Ecology lab of the Paris Lodron University of Salzburg. Compounds detected in similar amounts in leaf controls and flowers were excluded from the analyses. We only considered compounds that were present in more than twice the amount in flowers than in leaves. For quantitative analysis of VOCs, we injected 100 ng each of *ca.* 150 components, among them monoterpenes, aliphatic, and aromatic compounds, into the GC/MS system. We used the mean of the peak areas (total ion current) of these compounds to estimate the total amount of scent available in the scent samples (Etl et al., 2016).

#### Floral Resource

To check if florivory affected the amount of nectar, we bagged 80 floral buds 1 day before anthesis from 11 plants (*n* = 3–11 flowers/plant). We assigned each bud to one of two treatments: (i) intact buds, and (ii) buds previously naturally attacked by the main florivore. A single plant contained both treatments. Approximately 12 h after flower opening (*ca.* 18:00 h), we withdrew the accumulated nectar and measured its volume using 50 μl microcapillary tubes (Galetto and Bernardello, 2005).

### Effect of the Experimentally Simulated Floral Damages on Hummingbird Pollination

To obtain a large enough sample size to investigate the possible effects of floral damages on hummingbird pollination, we caused the damages manually. Additionally, by causing damages manually we could ensure that all the flowers presented to the hummingbirds showed a standard damage, which would be impossible to guarantee if we used flowers naturally damaged by florivores. However, experimental manipulations do not always incur in the same physiological responses as feeding by natural herbivores (Baldwin, 1990). Therefore, prior to performing this experiment, we controlled for possible effects of experimentally simulated floral damages on floral scent and floral nectar, which are traits that could display a different physiological response to mechanical simulation of damages than that triggered by florivores. For that, we removed the half-upper portion of the corolla tube, replicating the most severe damage caused by the most common natural florivore, a caterpillar (Figure 1D). Then, we collected scent samples from manually damaged flowers (*n* = 3 flowers from 3 plants) and compared them to undamaged flowers (*n* = 3 flowers from 3 plants) using the same method described in section 2.3.3. Moreover, we sampled nectar volume of manually damaged flowers (*n* = 38 flowers from 11 plants) and compared them to undamaged flowers (*n* = 49 flowers from 11 plants) using the same method described in section 2.3.4.

We found that experimentally damaged flowers did not differ in their scent from intact flowers [total amount: Pseudo-F_(1, 2)_ = 1.30; *p* = 0.43; relative amount: Pseudo-F_(1, 2)_ = 0.87; *p* = 0.52; presence/absence: Pseudo-F_(1, 2)_ = 0.9; *p* = 0.53], nor in nectar volume (*F* = 0.006; *p* = 0.9367). Therefore, in our study system, manually caused damages did not lead to physiological responses in terms of scent or nectar production.

Additionally, to avoid any possibility of pollination not performed by hummingbirds (Tunes and Guimarães, 2020), we performed the further experiment only with flowers that presented an accentuated approach herkogamy, with the anthers placed below the stigma. We randomly selected 33 plants of the population (3–14 flowers/plant). We submitted the flowers from those plants to one of two treatments: (i) control flowers that were not damaged (*n* = 97 flowers); and (ii) mechanically simulated floral damage, emulating the most serious damage recorded by the most common natural florivore, a caterpillar (*n* = 98 flowers; Figure 1D). Each plant contained both treatments in similar amount. We isolated pre-anthesis buds from floral visitors until our manipulations to guarantee that the flowers were all intact at the beginning of the experiment, and randomly assigned the flowers to the two treatments. To experimentally damage the flowers, we used surgical scissors to cut off pieces of the corollas according to the damages naturally caused by Lycaenidae caterpillars (Figure 1D). As we were interested in understanding the effects of florivory on plant–pollinator communication and pollination, we focused on corolla damage, because any damage to reproductive structures would definitely affect plant reproductive success. To ensure that control flowers remained intact, we checked these flowers at the beginning and the end of the experiment; flowers that had any damage at the end of the experiment were discarded and replaced in the subsequent days. We performed the experiments during the flowering peak, through 25 consecutive days, when pollinators and flowers were abundant (July–August). To evaluate the likelihood of a flower receiving hummingbird visits, we used two different and complementary approaches. Initially, we performed focal observations (in person and by video recording) of the pollinator visits (*n* = 8 plants, 30 h, 2–4 h of observation/plant/turn). We registered approximately two visits per plant per hour in the population at the peak of hummingbird visitation, which occurred from dawn until 09:00 h and from 17:00 until dusk. However, this approach did not work well, as visitation to the focal flowers was extremely low. This issue could be due to the fact that hummingbirds actively avoided patches where the observer was located. We needed to position ourselves and the cameras close to the plants (1.5–2 m from them) in order to clearly assess whether hummingbirds visited an intact or damaged flower. Thus, through this method, we could not obtain a robust data set to statistically compare the visits to each treatment.

To overcome this constraint, we additionally evaluated the presence of pollen deposited onto the stigmas of *P*. *venusta* flowers, which is a reliable proxy of a pollinator visit (Ashman et al., 2020). Therefore, we labeled 195 flowers (*n*_control_ = 97 flowers; *n*_damaged_ = 98 flowers) and exposed them to hummingbird visits during the 2 days of anthesis. Following corolla abscission, we collected the stigmas, fixed them in FPA solution (formalin 40%, concentrated propionic acid, ethanol 50%, in volumes of 5:5:90), and stained them with aniline blue and potassium acetate, following the protocol proposed by Dafni et al. (2005). Then, we evaluated the presence/absence of pollen grains adhered to the stigmas’ surfaces under a fluorescence microscope (Zeiss Axioskop 40 Microscope, with AxioVision 4.7.2) equipped with a filter set (of maximum transmission at 365 nm).

### Statistical Analysis

We used GLMM with gamma error distribution to evaluate if the removal of a portion of the corolla, due to florivory, lead to a change in the chromatic and achromatic contrasts of the anthers and the stigma against the remaining portion of the corolla, considering the interaction between type of reproductive structure (anthers or stigma) and treatment (before or after damage). Plant and flower were used as random variables. We performed a PERMANOVA (9,999 permutations) to evaluate if the total and relative amounts of floral scent as well as qualitative scent properties differed among treatments (control and damaged flowers), considering plant individual as a random factor, based on Euclidean distances for the total amount of scent, on Bray–Curtis dissimilarities for the relative amount of scent, and on Sørensen dissimilarities for qualitative scent properties (presence and absence of compounds). These three different scent characteristics were analysed as they all might influence floral visitor choices. We performed all PERMANOVA analyses using Primer 6 v. 6.1.15 with PERMANOVA+ v. 1.0.5. We verified if florivory affected nectar volume using GLMM with gamma error distribution, considering treatment as a fixed variable and individual plant as a random variable. We carried out both GLMMs with gamma error distribution in R v. 4.0.2 (R Core Team, 2020) with additional packages: car (Fox and Weisberg, 2019), emmeans (Lenth, 2020), fitdistrplus (Delignette-Muller and Dutang, 2015), ggplot2 (Wickham, 2016), glmmADMB (Fournier et al., 2012; Skaug et al., 2016), lattice (Sarkar, 2008), lme4 (Bates et al., 2015), MASS (Venables and Ripley, 2002), R2admb (Bolker et al., 2020), and viridis (Garnier, 2018). The probability of pollen grains (binary variable) being deposited onto the stigmas was modelled, using GLMM with binomial error distribution, again considering treatment as a fixed variable and individual plant as a random variable. This GLMM was carried out in R v. 4.0.2 (R Core Team, 2020) with standard and additional packages: lme4 (Bates et al., 2015), and nlme (Pinheiro et al., 2018).

## Results

### Natural Incidence of Florivory and the Florivores Associated to It

We sampled 935 flowers of *Pyrostegia venusta* (Bignoniaceae), from which 77.4% did not present any damage, while 22.6% showed variable degrees of corolla tissue removal. The most common damage corresponded to the category of 1–3% of corolla removal (registered in 14% of the flowers), followed by the 4–5% of corolla removal (registered in 3.7% of the flowers), then by the 6–10% of corolla removal (1.9% of the flowers) and by the other five categories, corresponding to larger damages, which comprised the remaining 3% of the flowers (less than 1% of the flowers per category).

**Figure 3:**
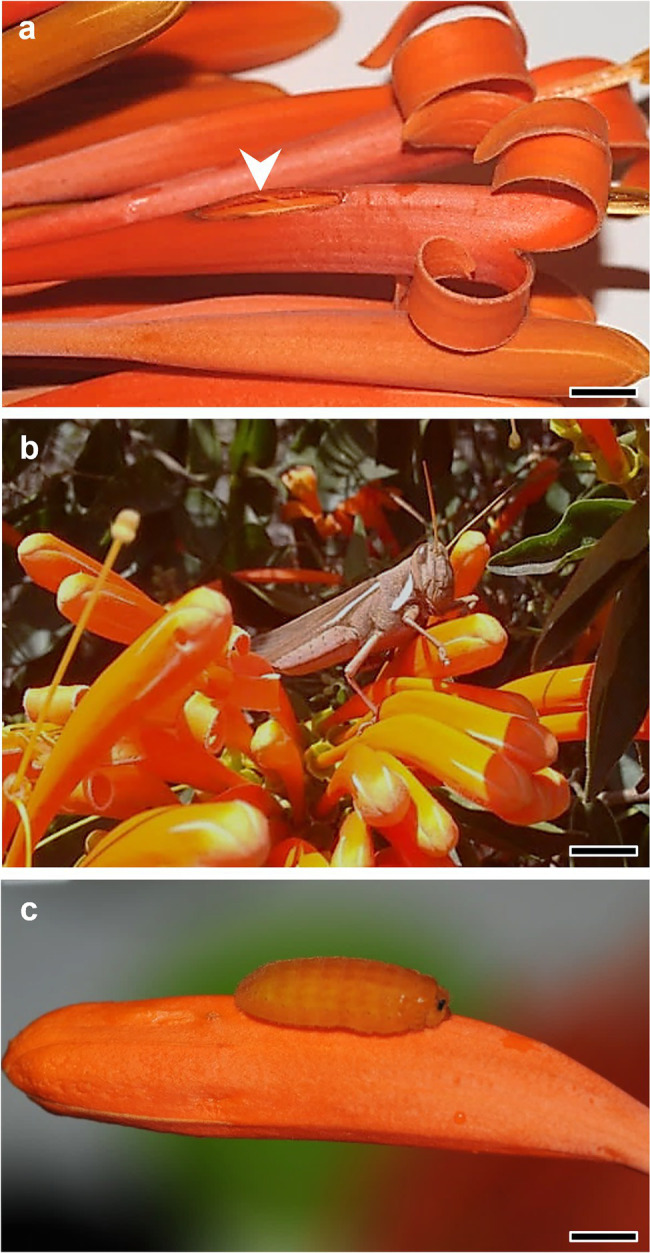
*Pyrostegia venusta* and its florivores. **(A)** Natural floral damage (arrowhead) with 1–3% of corolla damage. Scale bar: 0.7 cm. **(B)** *Schistocerca flavofasciata* (yellow-lined grasshopper, Acrididae) which consumed the flowers leaving bite marks as shown in Figure 1E. Scale bar: 1.3 cm. Photograph by N. M. Gildo. **(C)** *Parrhasius polibetes* (black-spot hairstreak, Lycaenidae) caterpillar, which was the most common florivore in *P*. *venusta*. Scale bar: 0.4 cm.

The grasshopper *Schistocerca flavofasciata* (yellow-lined grasshopper, Acrididae) consumed the medium dorsal portion of the corolla tube (Figures 3A,B) and small *Trigona* bees made holes in the corolla tube of floral buds, near the portion where the anthers were placed. Both florivores removed 1–3% of corolla tissues. Caterpillars of *Parrhasius polibetes* (black-spot hairstreak, Lycaenidae; Figure 3C) were the most common florivore, corresponding to *ca.* 90% of all the observed florivores in the field. They feed on the corolla and on floral reproductive structures. We observed in the field these caterpillars causing floral damages that belonged to every category, from the category of 1–3% until the category of 51–60% of corolla removal.

### Effect of Florivory on Floral Traits

#### Floral Outline

**Figure 4:**
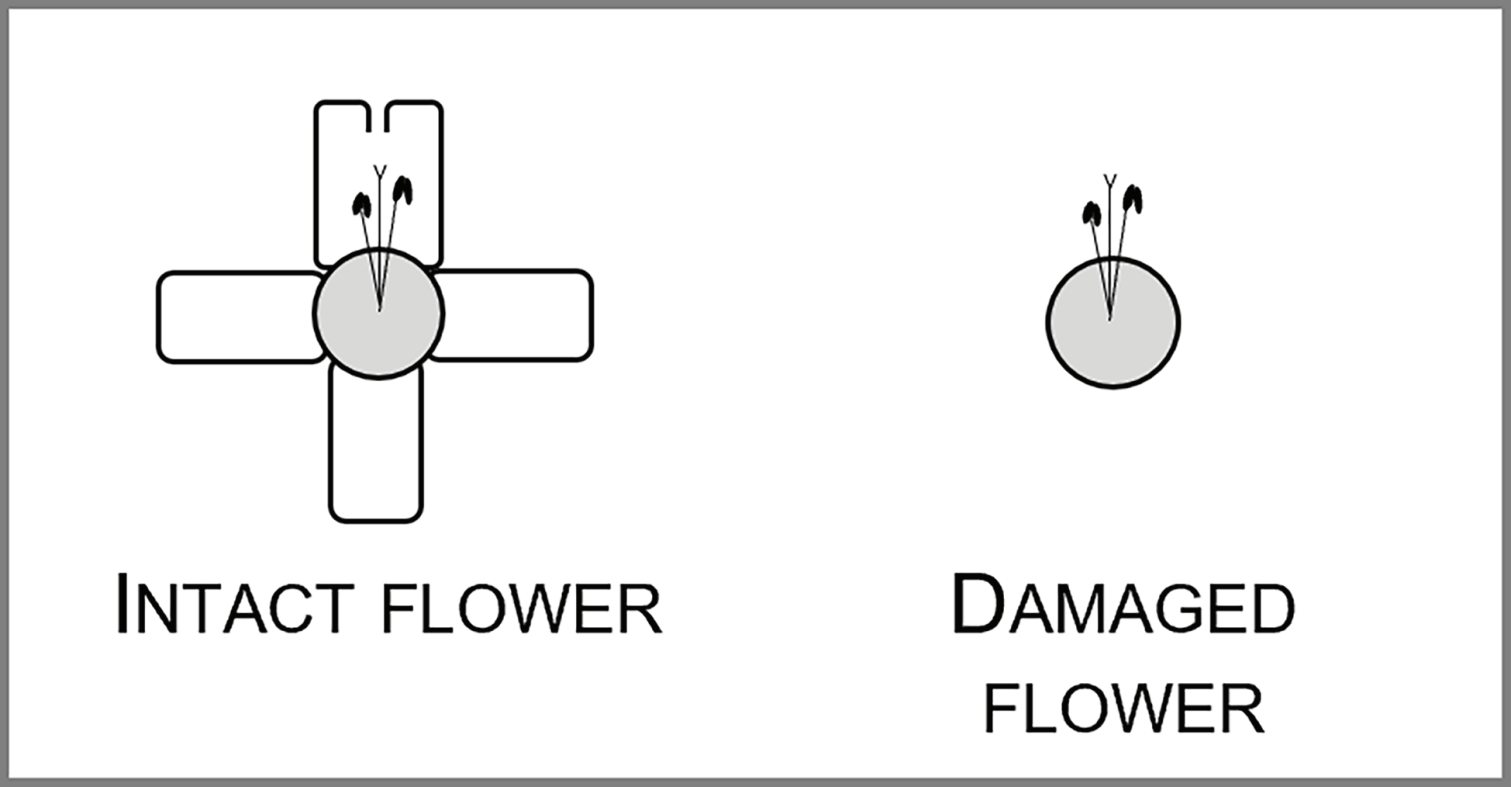
Schematic representation of the most pronounced damage caused by a lycaenid caterpillar florivore. The floral tube, the five corolla lobes, stamens and style are represented.

The only florivore that affected floral outline was the lycaenid caterpillar. Larvae of these butterflies consumed from small to large amounts of the corolla, including the lobes and upper portion of the corolla tube. Therefore, they changed the corolla outline, especially, when we consider the most pronounced levels of florivory, which led to the complete removal of the petal lobes in approximately 0.5% of the flowers (Figures 1D, 4).

#### Intra-floral Colour Pattern

**Figure 5:**
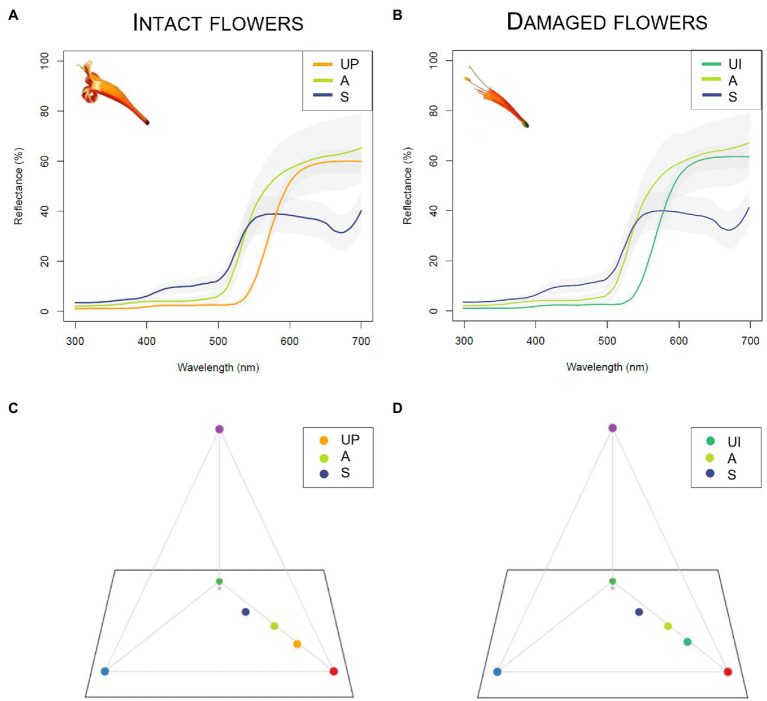
Spectral reflectance of intact and damaged *Pyrostegia venusta* (Bignoniaceae) flowers (*n* = 20 flowers from 8 plants) and colours modelled using the tetrahedron vision model for birds. **(A,B)** Spectral reflectance curves. The lines represent the mean reflectance of the upper petal lobe (UP), the upper internal portion of the corolla tube (UI), the anthers (A) and the stigma (S). The shaded areas represent the standard variation of reflectance measures. **(C,D)** Tetrahedron vision model for birds. The central grey dots represent the achromatic center; the coloured dots represent the mean loci for the aforementioned portions of the flowers. The maximum excitation of the ultraviolet, blue, green, and red photoreceptors is represented by the respective tetrahedron vertices (violet, blue, green, and red).

The flowers presented no UV reflection/absorption pattern and were UV-absorbing (Supplementary Figure 2). Therefore, the only intra-floral colour pattern present in these flowers was the human-visible pattern between the reproductive structures and the corolla. The chromatic and achromatic contrasts between the anthers/stigma and the corolla lobes in intact flowers are similar to the chromatic and achromatic contrasts between the anthers/stigma and the remaining lower half of the corolla in damaged flowers (Figure 5; *p* > 0.05; see Supplementary Table 1 for mean values and Supplementary Table 2 for statistical details).

#### Floral Scent

**Figure 6:**
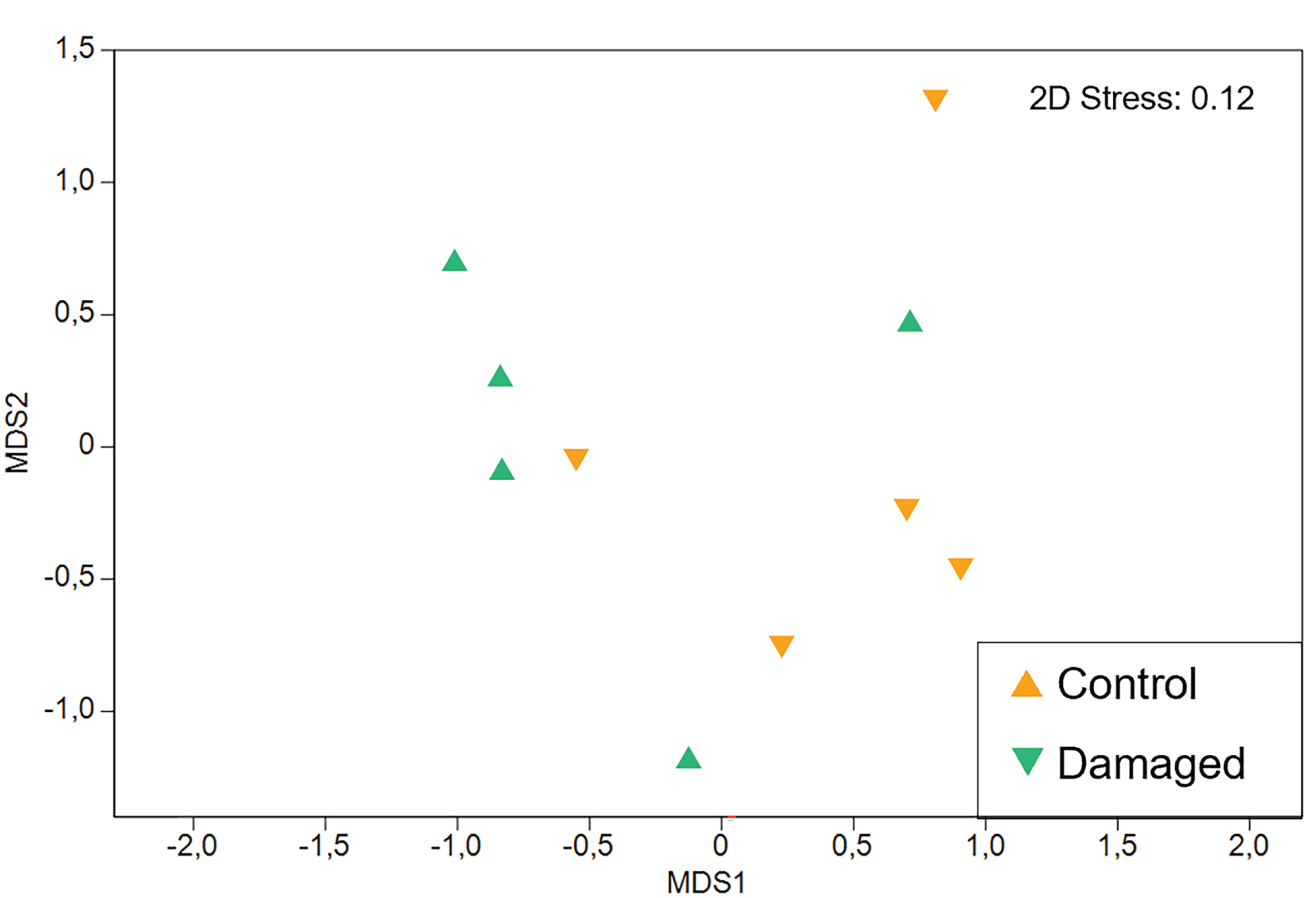
Non-metric multidimensional scaling (NMDS) of *Pyrostegia venusta* floral scent from control and naturally damaged flowers. Comparison between the relative scent composition emitted by flowers that were intact (control) or naturally damaged by the florivore caterpillar, *Parrhasius polibetes* (black-spot hairstreak, Lycaenidae). There was no significant difference in relative scent composition among flowers submitted to both treatments (PERMANOVA with 9,999 permutations, Pseudo-F _[1, 2.06]_ = 2.06; *p* = 0.20). Each dot represents a sample.

Intact flowers and flowers that were damaged by florivores (category of 51–60% of corolla removal) emitted a similar amount of scent [Pseudo-F_(1, 2.12)_ = 2.26; *p* = 0.30; Table 1] and had a similar qualitative scent pattern [Pseudo-F_(1, 2.06)_ = 3.84; *p* = 0.11]. Both treatments emitted mainly aromatic compounds and terpenoids. These compounds were emitted in similar relative amounts by flowers of the two treatments [PERMANOVA ‘Treatment’; Pseudo-F_(1, 2.06)_ = 2.06; *p* = 0.20; Figure 6]. On average, the most abundant compounds were β-bourbonene as well as β-caryophyllene in control flowers, benzaldehyde in damaged flowers, and 4-methylanisole in both treatments. It is noteworthy that relative scent properties were, independent of the treatment, highly variable among plants (Table 1; Figure 6).

**Table 1:** Total absolute (mean ± sd; minimum–maximum; ng. flower-1. hour-1) and relative amount of each compound (mean ± sd; minimum–maximum; %) of scent in intact and naturally damaged flowers of *Pyrostegia venusta*.

		**Intact flowers**	**Naturally damaged flowers**
**n**_**flowers**_ **(n**_**plants**_**)**		5 (3)	5 (5)
**Total number of compounds**		23	18
**Mean total amount of scent**		43.1 ± 39.80 (12.6–112.7)	30.1 ± 36.93 (2.7–94.4)
			
**Compound class**	**RI**		
**Aliphatic compounds**			
Hexanal*	798	2.37 ± 3.81 (0–8.76)	8.21 ± 12.58 (0–28.51)
(*E*)-2-Hexenal*	852	0.46 ± 0.65 (0–1.39)	-
**Aromatic compounds**			
Benzaldehyde*	963	8.46 ± 18.91 (0–42.3)	33.96 ± 31.85 (0–76.59)
4-Methylanisole*	1,025	12.03 ± 15.06 (0.82–37.6)	18.81 ± 29.04 (0.23–70.21)
Benzyl alcohol*	1,036	3.59 ± 8.04 (0–17.98)	2.79 ± 4.4 (0–10.31)
2-Phenylethanol*	1,118	1.13 ± 1.8 (0–4.14)	–
Methyl salicylate*	1,204	–	3.61 ± 7.25 (0–16.54)
**Terpenoids**			
6-Methyl-5-hepten-2-one*	987	4.25 ± 5.92 (0–12.15)	5.27 ± 5.52 (0–12.86)
Limonene*	1,036	2.26 ± 4.24 (0–9.76)	0.19 ± 0.42 (0–0.95)
α-Copaene*	1,395	3.31 ± 3.32 (0–7.53)	1.39 ± 3.12 (0–6.99)
β-Bourbonene*	1,407	16.35 ± 10.1 (0–26.75)	5.14 ± 11.51 (0–25.74)
β-Caryophyllene*	1,445	14.67 ± 30.22 (0–68.62)	6.41 ± 14.34 (0–32.07)
α-Caryophyllene*	1,481	5.03 ± 11.25 (0–25.16)	1.98 ± 4.42 (0–9.9)
Valencene*	1,515	4.12 ± 5.65 (0–10.62)	0.55 ± 1.23 (0–2.75)
(*E*)-Nerolidol*	1,571	0.52 ± 0.54 (0–1.27)	0.16 ± 0.35 (0–0.8)
1-*nor*-Bourbonanone	1,588	5.61 ± 5.5 (0–11.2)	4.72 ± 6.49 (0–12.58)
**Unknown compounds**			
m/z: 43.82.67.55.41.83	1,292	0.4 ± 0.55 (0–1.08)	–
m/z: 57.161.105.43.119.83	1,389	0.7 ± 0.97 (0–1.97)	–
m/z: 161.120.105.91.43.55	1,441	2.58 ± 1.54 (0–3.95)	0.99 ± 1.7 (0–3.93)
m/z: 161.105.91.119.79.133	1,452	2.35 ± 3.41 (0–7.47)	1.17 ± 2.62 (0–5.86)
m/z: 105.93.79.121.161.204	1,513	2.43 ± 5.44 (0–12.17)	3.44 ± 7.69 (0–17.2)
m/z: 41.121.55.206.163.93	1,638	1.96 ± 2.68 (0–5.11)	–
m/z: 43.108.93.126.41.71	1,706	4.35 ± 6.77 (0–15.44)	1.13 ± 1.83 (0–4.21)
m/z: 43.91.121.79.107.135	1,741	0.98 ± 1.57 (0–3.61)	–

Scent compounds are listed according to their chemical class and Kovats’ Retention Index (RI) based on a series of n-alkanes (C6–C20). Compounds marked by an asterisk were identified based on their mass spectra and the RI of synthetic standards.

#### Floral Resource

**Figure 7:**
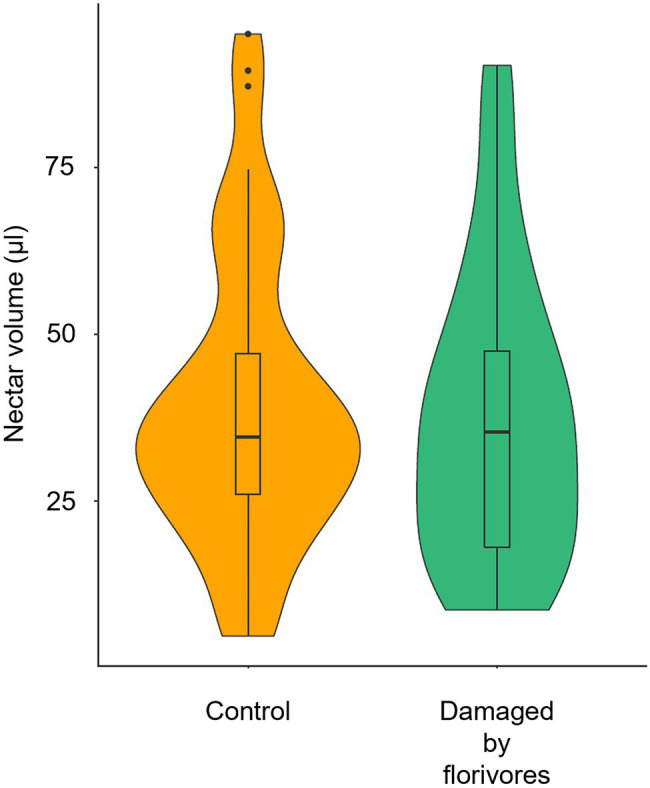
Violin plots of nectar volume in *Pyrostegia venusta* flowers that were intact (control) and damaged by florivores. The median, the 25th and 75th percentiles, the non-outlier range (within 1.5 times the interquartile range), and the outliers are shown. There was no significant difference among the nectar volume in flowers submitted to the different treatments (GLMM with gamma error distribution, *p* = 0.3384).

We found no difference when comparing control (39.2 ± 21.6 μl, mean ± sd) and naturally damaged (37.3 ± 21.8 μl) flowers regarding nectar volume (Figure 7; *F* = 0.916; *p* = 0.3384).

### The Effect of Experimentally Simulated Floral Damages on Hummingbird Pollination

Our beforementioned results evinced that natural florivory did not affect intra-floral colour pattern, floral scent, or nectar production. These results validate the use of mechanically damaged flowers (which did not alter those floral traits either) to evaluate the outcomes of florivory on hummingbird pollination.

We recorded the hummingbirds *Phaethornis pretrei* (Phaethornithinae), *Chlorostilbon lucidus* (Trochilinae), and *Polytmus guainumbi* (Trochilinae) as pollinators of *P*. *venusta*. All of them always contacted both male and female reproductive structures while visiting control and damaged flowers. We did not observe a difference when comparing the likelihood of pollen grains being deposited onto the stigmas of intact and damaged flowers (*Z* = 0.279, *p* > 0.1, *n*_flowers_ = 195, *n*_plants_ = 33), with 47.07 ± 74.37 (mean ± sd) pollen grains found in the stigmas of control flowers and 44.56 ± 75.22 in damaged flowers.

## Discussion

**Figure 8:**
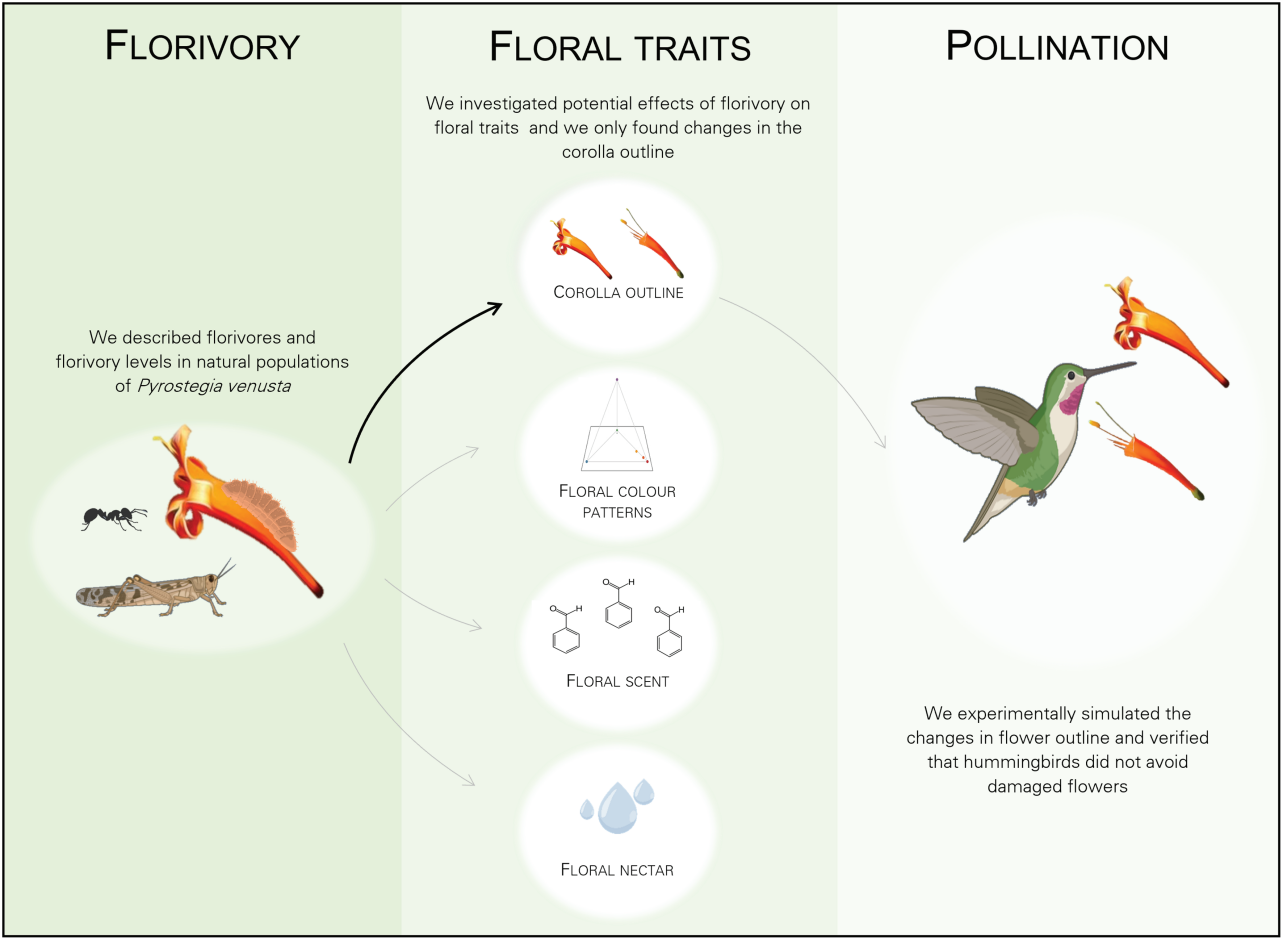
Graphical summary of the experimental design of the study showing the general design and main results. Florivory, by the most common florivore, a Lycaenidae caterpillar, did not affect floral colour patterns, nor floral scent, nor floral nectar volume (grey arrows). Florivory only affected flower integrity, by changing the corolla outline due to the presence of damages *per se* (black arrow). Finally, we show that there was no effect of the mechanical simulation of floral damages on pollination (grey arrow). Created with BioRender.com.

In natural populations, the majority of *P*. *venusta* flowers (77.4%) was intact and the remaining 22.6% of the flowers showed variable natural levels of floral damage, being a Lycaenidae caterpillar the most frequent florivore observed in the system. This florivore caused damages, which were variable in size and shape, fitting the categories from 1–3% to 51–60% of corolla removal. Despite the accentuated loss of corolla integrity caused by this florivore, floral colour patterns, floral scent locally emitted by damaged flowers and floral nectar volume were not affected. Mechanical simulation of the change in corolla outline caused by caterpillars did not affect pollination by hummingbirds (see a graphical summary of the main findings of this study in Figure 8).

*Pyrostegia venusta* presents a massive visual floral display, with synchronous production of numerous orange short-lived flowers (Gobatto-Rodrigues and Stort, 1992). This massive visual advertisement will not only be perceived by pollinators, but also by florivores (Anderson, 1996; Harder and Johnson, 2005). Indeed, not only hummingbirds, but also grasshoppers and butterflies (which lay eggs inside the flowers that develop into caterpillar florivores), are capable of perceiving orange flowers (Vishnevskaia and Mazokhin-Porshniakov, 1969; Chittka and Briscoe, 2001 and references therein; Vanhoutte, 2003; Briscoe, 2008). Thus, one can expect high levels of florivory due to the local abundance of resources. Similarly, to our study system, Kessler et al. (2013) also found a prevalence of natural low levels of floral tissue removal performed by orthopterans. High levels of florivory, comparable in size to the largest damages observed here, also performed by lepidopteran caterpillars, were described for a bee-pollinated system (McCall, 2008).

Regarding floral visual advertisements, we could expect at least two types of effect of florivory. The first and more obvious one is the loss of the physical integrity of the corolla. Although after florivory the remaining portion of the corolla is still tubular, the general outline of the flower was modified. Hummingbirds perceive floral shapes and those with short straight bill prefer shorter and straighter floral tubes, whereas hummingbirds with long curved bills prefer longer and more curved flowers (Maglianesi et al., 2015). Therefore, as hummingbirds present this capability of distinguishing floral shapes, especially regarding floral tube length, the damages caused by florivores could lead hummingbirds not to recognise the ‘new’ floral outline as that originally associated with the trophic resource (intact flowers). Nevertheless, contrary to this expectation, our results show that hummingbirds did not reject damaged flowers. A second expected effect of florivory on floral visual advertisements is changes in intra-floral colour pattern. Florivory did not affect intra-floral colour patterns, as the chromatic and achromatic contrasts between the yellowish reproductive structures and the orange corolla did not differ between intact and damaged flowers. Overall, florivory in *P*. *venusta* changed floral outline but preserved intra-floral colour patterns, with no effects on pollination success. This contrasts with the findings for *Mimulus luteus*, another hummingbird-pollinated species, in which florivory changed colour patterns, with negative effects on pollination (Pohl et al., 2006).

Regarding floral chemical advertisements, hummingbird-pollinated flowers are commonly regarded as being scentless or present few and widespread VOCs (Knudsen et al., 2004). However, we found 24 scent compounds in *P*. *venusta*, which is up to 8.3 times greater than what had been previously described for other hummingbird-pollinated species (Knudsen et al., 2004). Among these compounds are several that are also known from other hummingbird-pollinated plants (Knudsen et al., 2004; Dellinger et al., 2019). Some are even behaviorally active in hummingbird species [attractants: benzaldehyde, (E)-β-caryophyllene; repellents: limonene, methyl salicylate; see Kessler and Baldwin (2007)] and might also be involved in the communication between *P*. *venusta* and their hummingbird pollinators. Similarly to the number of compounds, also the total amount of floral scent is highly variable among hummingbird-pollinated species, also showing high levels of intra-specific variation (Dellinger et al., 2019), as we recorded in *P*. *venusta*. The ecological consequences of intra-specific scent variation in hummingbird-pollinated species are unknown so far, but are currently under investigation (Guimarães et al., unpublished data). Regardless, as the amount of scent emitted by *P*. *venusta* flowers and its relative chemical composition were similar in intact and damaged flowers, one can infer that both types of flowers, based on the olfactory advertisement, are indistinguishable to hummingbirds.

Although visual and chemical advertisements are determinant for flower location by hummingbirds, a reliable nectar supply will be essential for maintaining hummingbird visits to the flowers (George, 1980; Irwin, 2000; Altshuler and Nunn, 2001; Hurly and Healy, 2002; Chautá et al., 2017). Damages inflicted by florivores to *P*. *venusta* flowers did not affect the amount of nectar available per flower, differently from shown by Radhika et al. (2010) for *Brassica napus*. Therefore, by operant conditioning, with nectar acting as positive reinforcement, hummingbirds can easily learn to keep visiting *P*. *venusta* flowers, even in the presence of severe damages.

In general, most studies that evaluated the effect of florivory on plant fitness have found negative effects; however, this result is vastly variable among plant species (see Haas and Lortie, 2020 for references). These negative effects are associated with pollen limitation (Leavitt and Robertson, 2006; McCall, 2010), or a specific decrease in plant attraction to bees (Karban and Strauss, 1993; Krupnick et al., 1999; McCall, 2008), bats (Von Helversen and Von Helversen, 1999) and hawkmoths (Mothershead and Marquis, 2000). Regarding hummingbird-pollinated systems, the studies have shown contrasting results. Our experimental approach revealed that florivory did not affect *P*. *venusta* pollination, as found by Tsuji et al. (2016). However, in other systems, it negatively altered plant fitness, especially, when florivory affected floral guides (Pohl et al., 2006). It is possible that the mechanisms underlying these variable outcomes, which are poorly known, may be species-specific from plant, pollinator, and florivore perspectives (Rusman et al., 2019b).

Moreover, the ecological consequences of florivore-induced local short-term changes on plant–pollinator interactions may be substantially distinct from those of systemic responses. Some of the effects of florivory are only expressed locally, such as changes in flower shape, outline and size, or changes in the intra-floral colour patterns, as direct ‘byproducts of florivore action.’ However, other effects, such as volatile emission, pigment allocation, and nectar features, can change due to a local or a systemic response, as a ‘plant reaction to florivore action.’ Local effects of florivory should be fast enough to be expressed during the flower lifetime. On the other hand, systemic effects may be fast or not and will comprise the whole plant, including damaged and undamaged flowers (Rusman et al., 2019a). In our study, we found no differences in most of the traits evaluated, except for the inevitable change in the flower outline due to the removal of corolla portions by the florivores, which is a local direct effect of florivory. From the pollinator perspective, the flower is the unit that must be recognised when searching for resources. Thus, the short-term local effects of florivory have a large potential to immediately interfere with flower–pollinator communication pathways.

There is a growing body of literature regarding the effects of florivory on pollination (Soper Gorden and Adler, 2016, see Haas and Lortie, 2020 for references) and the present study simultaneously investigated the local effects of florivory on visual and olfactory advertisements, as well as on floral sugar resource. Considering all our data, we have shown that florivory only led to the loss of corolla integrity due to the presence of damages *per se*, which changed corolla outline. However, during the flower lifespan, florivory did not have effects on intra-floral colour patterns, floral scent, or floral nectar. Thus, this study isolates a single effect of florivory—floral outline modification—and demonstrates that this isolated change in flower appearance does not discourage hummingbird visitation, even though hummingbirds strongly rely on vision for food location (Hickman and Robert, 1993; Pritchard et al., 2017; Tyrrell et al., 2018 and references therein). Thus, this study highlights that the florivore–plant–pollinator intersection may work as a complex and stable trophic system, in which local changes in floral traits promoted by florivory are not enough to disrupt flower–hummingbird communication and pollen transfer itself.

## Data Availability Statement

The raw data supporting the conclusions of this article will be made available by the authors upon request, without undue reservation.

## Author Contributions

PT and EG conceived the study, collected and analysed the data, and wrote the main manuscript text. SD contributed to the scent analyses and commented on earlier versions of the manuscript. All authors contributed critically to the manuscript and approved the final version.

## Funding

This work was supported by the São Paulo Research Foundation (FAPESP), Grant #2018/14146–0 and Grant #2009/17611–7 and by the National Council of Technological and Scientific Development, Proc. 312799/2021-7 to EG. This study was financed in part by the Coordination of Superior Level Staff Improvement, Brazil, Finance Code 001 and PDSE #88882.433249/2018–01 to PT.

## Conflict of Interest

The authors declare that the research was conducted in the absence of any commercial or financial relationships that could be construed as a potential conflict of interest.

## Publisher’s Note

All claims expressed in this article are solely those of the authors and do not necessarily represent those of their affiliated organizations, or those of the publisher, the editors and the reviewers. Any product that may be evaluated in this article, or claim that may be made by its manufacturer, is not guaranteed or endorsed by the publisher.
