# Supplementary material for: Florivory and Pollination Intersection: Changes in Floral Trait Expression Do Not Discourage Hummingbird Pollination
Source: Front Plant Sci. 2022 Mar 30;13:813418. doi: 10.3389/fpls.2022.813418 (PMC9006511; doi:10.3389/fpls.2022.813418)
Supplement: Supplementary file 1 [file Data_Sheet_1.docx]

Supplementary Material


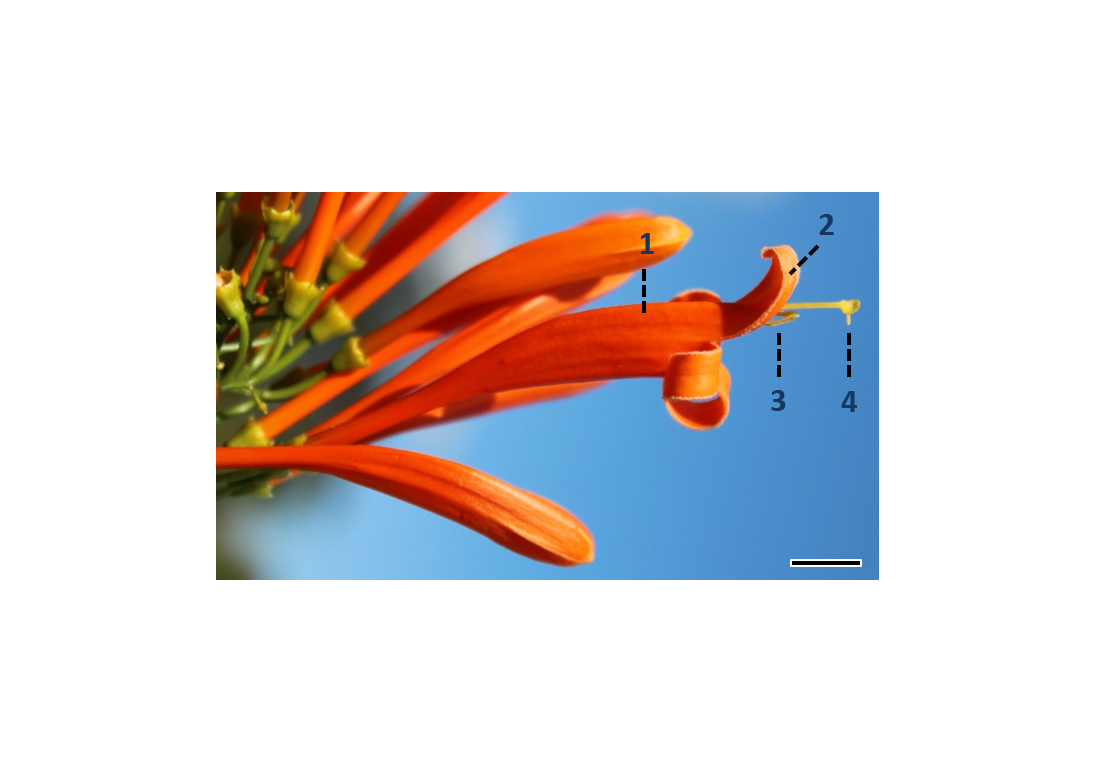


**Supplementary Figure 1.** **Floral portions of *Pyrostegia* *venusta* in which we measured the spectral reflectance.** 1: upper internal portion of the corolla tube (UI); 2: upper petal lobe (UP); 3: anthers (A); 4: stigma (S). Scale bar: 0.9 cm.


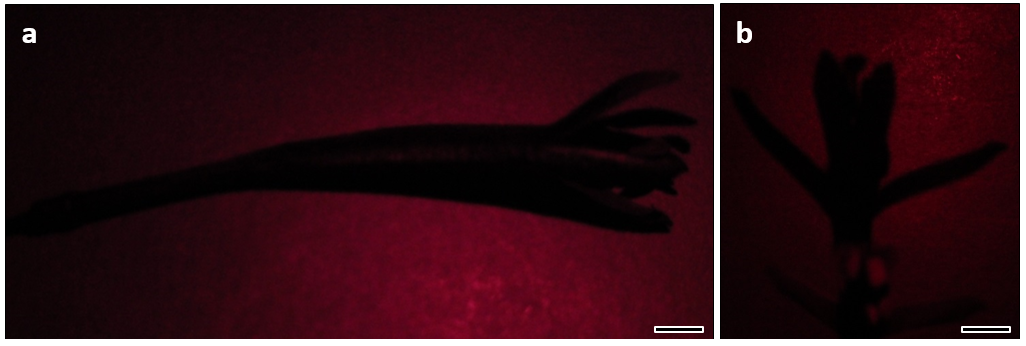


**Supplementary Figure 2.** ***Pyrostegia venusta* (Bignoniaceae) flower photographed using a camera with a modified sensor that only captures UV light and illuminated by an UV light source. Surfaces that reflect UV light become bright red in the image.** **(A)** Side view; **(B)** Frontal view. Scale bar: 0.5 cm. Note that, in both views of the flower, we cannot observe any portion of the flower in red, which indicates that the whole flower absorbs UV-light.

**Supplementary Table 1.** Mean and standard deviation of the chromatic and achromatic contrasts between floral parts in Just Noticeable Differences (JNDs) of *Pyrostegia venusta* flowers (n = 20 flowers from eight plants). The contrasts of both reproductive structures against the upper petals correspond to the contrasts observed in intact flowers, and the contrasts against the upper internal portion of the corolla tube correspond to the contrasts observed in damaged flowers.

| **Treatment** | **Contrasting floral parts** | **Chromatic contrast** | **Achromatic contrast** |
| --- | --- | --- | --- |
| Control | Anthers - Upper petal lobes | 6,32 ± 2,06 | 3,84 ± 2,22 |
|  | Stigma - Upper petal lobes | 12,07 ± 2,13 | 2,6 ± 1,74 |
| Damaged | Anthers - Upper internal portion of the corolla tube | 5,68 ± 1,72 | 3,7 ± 2,02 |
|  | Stigma - Upper internal portion of the corolla tube | 11,23 ± 2,55 | 2,42 ± 1,8 |

**Supplementary Table 2.** GLMM results, based on the models “chromatic contrast ~ treatment + contrasting parts + (1|plant) + (1|flower)” and “achromatic contrast ~ treatment + contrasting parts + (1|plant) + (1|flower)”. A = anthers, S = stigma, SE = standard error.

| Type of Contrast | ‘Treatment’ effect | | | | | ‘Contrasting parts’ effect | | | | |
| --- | --- | --- | --- | --- | --- | --- | --- | --- | --- | --- |
|  | Comparison | Coefficient | SE | Z value | p-value | Comparison | Coefficient | SE | Z value | p-value |
| Chromatic | A Control-Damaged | -0.0085 | 0.0053 | -1.595 | 0.1107 | Control A-S | 0.0797 | 0.0066 | 12.166 | **<.0001** |
|  | S Control-Damaged | -0.0085 | 0.0053 | -1.595 | 0.1107 | Damaged A-S | 0.0797 | 0.0066 | 12.166 | **<.0001** |
| Achromatic | A Control-Damaged | -0.0147 | 0.0497 | -0.296 | 0. 7670 | Control A-S | -0.1283 | 0.0543 | -2.364 | 0. 0181 |
|  | S Control-Damaged | -0.0147 | 0.0497 | -0.296 | 0.7670 | Damaged A-S | -0.1283 | 0.0543 | -2.364 | 0.0181 |
